# Draft whole-genome sequences of three *Borrelia burgdorferi* isolates from Western Canada

**DOI:** 10.1128/mra.00879-23

**Published:** 2024-01-05

**Authors:** Thomas H. Haidl, Min-Kuang Lee, Sean D. Workman, Jennifer N. Russell, Erin Fraser, Muhammad G. Morshed, Andrew D. S. Cameron

**Affiliations:** 1 Institute for Microbial Systems and Society, Faculty of Science, University of Regina, Regina, Saskatchewan, Canada; 2 Department of Biology, Faculty of Science, University of Regina, Regina, Saskatchewan, Canada; 3 Zoonotic Diseases & Emerging Pathogens, BC Centre for Disease Control-Public Health Laboratory, Vancouver, British Columbia, Canada; 4 BC Centre for Disease Control, Vancouver, British Columbia, Canada; 5 School of Population & Public Health, University of British Columbia, Vancouver, British Columbia, Canada; 6 Department of Pathology and Laboratory Medicine, University of British Columbia, Vancouver, British Columbia, Canada; Indiana University, Bloomington, Indiana, USA

**Keywords:** *Borrelia burgdorferi*, Lyme disease, *Ixodes pacificus*

## Abstract

Whole-genome sequences are presented for three *Borrelia burgdorferi*, a causative agent of Lyme disease in North America, isolated from *Ixodes pacificus* ticks collected in British Columbia, Canada. Shotgun DNA libraries were prepared with Illumina DNA Prep and sequenced using the MiniSeq platform. Genome assemblies enabled multilocus sequence typing and *ospC* typing.

## ANNOUNCEMENT

*Borrelia burgdorferi* sensu stricto is the predominant causative agent of Lyme disease in North America (1), the most common vector-borne disease in the region (1–3). In Canada, the highest disease risk is associated with the black-legged tick *Ixodes scapularis* in central to eastern provinces (4–7). *B. burgdorferi* is endemic but less prevalent in southern British Columbia, where it is spread primarily by the tick *Ixodes pacificus* (4). To investigate *B. burgdorferi* genome composition in Western Canada, we sequenced the whole genomes of isolates 21T 0066, 22T 0045, and 22T 0109.

The strains were cultured from three *I. pacificus* ticks found on humans. Strains 21T 0066 and 22T 0045 were isolated from ticks collected in Victoria (2021 and 2022, respectively), and strain 22T 0109 was isolated from a tick collected on Galiano Island (2022). Ticks were submitted to the British Columbia Center for Disease Control, surface disinfected using 10% hydrogen peroxide and 70% ethanol, and then dissected. The contents of their midgut were cultured at 34°C in modified Barbour-Stonner-Kelly medium containing kanamycin and rifampicin at concentrations of 10 µg/mL and 50 µg/mL, respectively. Cultures were checked weekly for spirochetes using dark-field microscopy, followed by quantitative PCR confirmation of *B. burgdorferi* using primers and probe for 23S rDNA, as previously described (8). Culturing was performed for periods up to 35 days, and positive cultures were then stored at −80°C before being shipped overnight on −80°C ice packs. Total genomic DNA was isolated from culture aliquots using the DNeasy UltraClean Microbial kit (Qiagen, Venlo, Netherlands). DNA extracts were quantified using the Qubit 2.0 fluorometer and dsDNA High Sensitivity assay kit (Thermo Fisher, Waltham, MA).

DNA sequencing libraries were prepared using Illumina DNA Prep (Illumina, CA, USA) and assumed to have an average fragment size of 600 bp. Final libraries were quantified using the NEBNext Library Quant Kit for Illumina (New England BioLabs; Ipswich, MA, USA) then normalized to 10 nM prior to pooling, diluting, and denaturing. Sequencing was performed on the Illumina MiniSeq platform using 300-cycle paired-end chemistry.

Sequence assembly was conducted entirely within BV-BRC Comprehensive Genome Analysis (9); default parameters were used at all stages of the workflow except where otherwise noted. Raw sequencing data were uploaded to BV-BRC in fastq format, and then Trim Galore (0.6.5dev) removed adapters and low-quality sequence per the “true” trim setting (10). Contigs were assembled by Unicycler (v0.4.8) (11) and then were filtered by default minimum thresholds of 300-bp length and fivefold coverage, followed by two polishing iterations using PILON (v1.23) (12). Assembly quality was assessed using QUAST (v5.2.0) (13). Genomes were subsequently annotated by the NCBI Prokaryotic Genome Annotation Pipeline (v6.5) (14); completeness assessed by CheckM (v1.2.2) (15) ranged from 99.4% to 99.7%.

Multilocus sequence typing (MLST) was performed using the PubMLST database (16), classifying isolate 21T 0066 as sequence type (ST) 13 and 22T 0045 as ST6. 22T 0109 matched ST6 at all MLST positions except for a single nucleotide variant 250G>A at the *nifS* locus, making 22T 0109 nearest ST6. The *ospC* gene encodes the primary surface antigen under immune selection pressure and so demonstrates high genetic diversity (17). Assemblies were queried against *ospC* reference sequences as in reference (18) using the BLASTn function in BLAST+ (v2.9.0) (19) and a >92.0% nucleotide identity threshold (20). 21T 0066 is *ospC* type M, while both 22T 0045 and 22T 0109 are *ospC* type H. Sequencing, assembly, genome quality, and annotation metrics for the three isolates are presented in Table 1.

**TABLE 1:** Sequencing, assembly, quality, and annotation metrics for *Borrelia burgdorferi* isolates

Isolate name	No. of paired reads	No. of bases	Average read length	Coverage (×)	No. of contigs	N50 contig size (bp)	Total size (bp)	Assembly G + C content (%)	No. of CDS	No. of RNAs	No. of pseudo genes	Multilocus sequence type	*ospC* type	Genbank accession no.	SRA Accession no.
21T 0066	504,431	151,329,300	150	83.2	70	894,843	1,223,151	28.6	1,210	41	32	13	M	JAUPGQ000000000	SRR25420533
22T 0045	427,022	128,106,600	150	84.4	124	213,028	1,249,516	28.1	1,251	38	28	6	H	JAUPGP000000000	SRR25420532
22T 0109	230,533	69,159,900	150	46.5	124	164,193	1,234,772	28.2	1,233	40	28	6^*a*^	H	JAUPGO000000000	SRR25420531

^*a*^ Isolate 22T 0109 was nearest sequence type 6. Seven of the eight loci were perfect matches, but *nifS* differed from the *nifS* sequence type 8 at 250G>A.

## Data Availability

This whole-genome sequencing project has been documented as NCBI BioProject PRJNA998043. Under this BioProject, sequence assemblies have been deposited in GenBank, and raw sequence read files have been uploaded to the NCBI sequence Read Archive (SRA). Accession numbers to these submissions for each isolate can be found in Table 1.
